# Adverse Experiences in Early Childhood and Emotional Behavioral Problems Among Chinese Preschoolers: Psychological Resilience and Problematic Media Use

**DOI:** 10.3390/bs15070898

**Published:** 2025-07-01

**Authors:** Yantong Zhu, Liu Yang, Gengli Zhang

**Affiliations:** 1 Faculty of Educational Science, Anhui Normal University, Wuhu 241000, China; ytzhu@ahnu.edu.cn; 2 School of Educational Science, Anhui Normal University, Wuhu 241000, China; yl6475264@gmail.com

**Keywords:** adverse childhood experiences, emotional behavioral problems, psychological resilience, problematic media use, preschool children

## Abstract

Adverse childhood experiences (ACEs) in early childhood may increase the risk of emotional behavioral problems (EBPs); however, few studies have explored the longitudinal effect of ACEs in early childhood on later EBPs and their underlying mechanisms. This study examined the serial mediating role of psychological resilience (PR) and problematic media use (PMU) in the relationship between ACEs in early childhood and EBPs. Participants included 534 three-year-old children (mean age of 33.29 years, SD = 3.97) and their parents from Wuhu, China. The parents completed online questionnaires measuring children’s ACEs, PR, and demographic information in September 2022 (Time 1), children’s PMU in September 2023 (Time 2), and EBPs in September 2024 (Time 3). Macro Process 6 was used to test the serial mediating effects of PR and PMU. A total of 5000 bootstrap samples were used to estimate the 95% confidence intervals. The results revealed that ACEs in early childhood did not directly predict EPBs. PR (b = 0.132 (95% CI [0.051, 0.221])) mediated the relationship between ACEs and children’s EBPs. PR and PMU also played a serial mediating role in the association of ACEs and children’s EBPs (b = 0.026, 95% CI [0.008, 0.054]). Our findings highlight the importance of improving PR and addressing PMU when designing interventions targeting Chinese preschoolers exposed to ACEs.

## 1. Introduction

Adverse childhood experiences (ACEs) refer to traumatic events that occur before the age of 18, including various forms of abuse and neglect (such as sexual, physical, or emotional abuse and neglect) and household dysfunction (such as witnessing domestic violence, experiencing parental divorce or separation, parental incarceration, or substance abuse in the household) (Felitti et al., 1998). A US national survey found that fifty-five kindergarten children are exposed to at least one adverse experience (Jimenez et al., 2016). Early childhood adversity has a significant impact, with toxic stress altering brain development and negatively influencing the simultaneous maturation of other organ systems and regulatory functions (Shonkoff et al., 2012). Studies have revealed that exposure to ACEs is related to learning difficulties and social–emotional and behavioral problems in young children (Loveday et al., 2022). Although studies have suggested the relationships between early adverse experiences and emotional behavioral problems (EBPs) in young children (Bright & Thompson, 2018; Webster, 2022), few studies have explored the longitudinal relations and underlying mechanisms among Chinese preschool samples. In traditional Chinese culture, authoritarian parenting styles and corporal punishment may be more socially accepted and even considered normative (Lansford et al., 2005). Parenting behaviors such as harsh disciplines that might be classified as “abuse” or “neglect” in Western culture may be perceived as discipline or guidance in Chinese contexts. As a results, Chinese children may internalize or interpret ACEs differently, which could modulate their psychological impact (Zhu et al., 2024). Studying the impacts of ACEs in a Chinese context is critical since the results may be affected by traditional culture (e.g., Confucian philosophies), and they may differ from those in Western countries.

### 1.1. ACEs and Emotional Behavioral Problems in Young Children

Young children are disproportionately exposed to traumatic events compared to older children (Lieberman et al., 2011), putting them at risk for poor short- and long-term developmental outcomes (Liming & Grube, 2018). Social–ecological theory posits that stressful childhood environments and events, as well as caregivers’ characteristics and functioning, have a negative impact on early childhood and late life development (Bronfenbrenner, 1979; J. K. Choi et al., 2019). According to stress sensitization theory, repetitive stress in early life disrupts stress response systems and lowers the threshold for reactivity and adaptive responses to later stress (Manyema et al., 2018), increasing the risk of developing mental health disorders (Nurius et al., 2013). Empirical research has revealed that more ACEs were associated with more attention problems, social problems, aggressive behaviors, chronic medical conditions, and developmental difficulties among Western samples (Kerker et al., 2015; Jimenez et al., 2016; Webster, 2022). More ACEs were also associated with more emotional problems in Chinese preschoolers (Zhu et al., 2022). However, few studies using longitudinal research have examined the association of ACEs with EBPs in young children and their underlying mechanisms.

### 1.2. The Mediating Role of Psychological Resilience

Psychological resilience (PR) refers to a personality trait, a dynamic process, or an outcome that enables individuals to adapt positively through continuous self-adjustment in the face of stress and adversity (Campbell-Sills et al., 2006). Previous studies have identified PR as a key intraindividual factor that mediates the relationship between ACEs and EBPs in children (Qiao et al., 2024). A meta-analysis by Morgan et al. (2022) reported that individuals exposed to ACEs often exhibit lower levels of PR. Similarly, previous research found that childhood adversities weakened resilience in both Chinese and Western samples (Yu et al., 2022; Shahidi et al., 2024). Furthermore, a lack of resilience has been linked to an increased risk of EBPs. Arslan (2016) highlighted that insufficient PR may contribute to the development of mental health problems. In contrast, individuals who effectively cope with adversity or risk—demonstrating resilience—are more likely to exhibit fewer emotional and behavioral difficulties (Rutter, 2006; Goldstein et al., 2013). Supporting this, C. C. Huang et al. (2019) found that resilience was negatively associated with EBPs among Chinese adolescents. Zhu et al. (2022) found that ACEs can decrease the level of PR and thus increase the risk of emotional problems among Chinese preschoolers. Taken together, these findings suggest that PR may serve as a critical mediator between early childhood adversities and the development of EBPs in children.

### 1.3. The Mediating Role of Problematic Media Use

A recent meta-analysis showed that the use of digital screen media has increased significantly across all age groups and at increasingly younger ages (Rega et al., 2023). By June 2023, there were 1.079 billion Internet users in China, with 3.8% of them being younger than ten years old (CNNIC, 2023). Problematic media use (PMU) refers to excessive use of different screen media devices (e.g., computer, videogames, smartphone, tablet, and television) that interferes with a child’s social, behavioral, and academic functioning (Domoff et al., 2020). When the utilization of media interferes with important facets of a child’s and adolescent’s life, it can lead to poor mental health and symptomatic behaviors such as difficulties ending media use, annoyance when unable to use media, and thoughts centered on device use (Domoff et al., 2019). Despite societal efforts to reduce screen time, these PMU behaviors are more predictive of psychosocial problems than screen time itself (Domoff et al., 2019).

Media Dependency Theory suggests that ACEs reduce children’s access to real-world support systems (e.g., caregivers), increasing dependence on media for distraction or comfort (Ball-Rokeach & DeFleur, 1976). As dependency grows, problematic media use emerges, worsening emotional and behavioral outcomes. Research suggests that early adversities can create cognitive–affective vulnerabilities, thereby increasing susceptibility to PMU (Schimmenti et al., 2017). Individuals who have experienced adversity may misuse media as a coping strategy (Worsley et al., 2018). Furthermore, previous studies indicated that PMU could increase children’s EBPs (Li et al., 2024). More specifically, children who use the Internet maladaptively and screens excessively often exhibit more internalizing and externalizing problems (Takahashi et al., 2018; Hussain et al., 2017; Manganello & Taylor, 2009). Based on previous theories and research, PMU may mediate the association between early childhood adversities and children’s EBPs.

### 1.4. The Serial Mediation Effect of Psychological Resilience and Problematic Media Use

According to The Interactional Theory of Childhood Problematic Media Use (IT-CPU; Domoff et al., 2020), distal factors are the aspects of a child’s developmental environment that may increase susceptibility to the development of PMU, while proximal factors are the specific antecedents of PMU behavior. ACEs in early childhood may be the distal factor that increase the development of PMU in children, which results in a lack of PR. As a result, children may use media maladaptively. Compensatory Internet Use Theory suggests individuals use media to compensate for unmet emotional or psychological needs (Kardefelt-Winther, 2014). Children with lower resilience may turn to excessive or compulsive media use to escape or regulate negative effects; PMU thus becomes a maladaptive coping strategy in the absence of PR (Hao et al., 2023). Empirical studies also showed the negative associations between PR and PMU (Hidalgo-Fuentes et al., 2023). A meta-analysis identified resilience as a mediator that can buffer the effect of harm (Sage et al., 2021). Cao et al. (2022) found that PR negatively correlated with Internet addiction among “left-behind” children in China. However, few studies have explored the serial mediation effect of PR and PMU in the relationship between ACEs and EBPs among Chinese preschool children.

### 1.5. Current Study

This study expects to explore the relationship between ACEs in early childhood and EBPs and their underlying mechanisms from a longitudinal perspective with three timepoints. Based on the relevant theories and empirical studies reviewed above, the current study focused on two research questions. The first research aim was to explore whether ACEs in early childhood predicted children’s EBPs two years later. Our second research question aimed to reveal whether PR and PMU played a serial mediating role in the relationship between ACEs and EBPs. By revealing the underlying mechanisms, the findings aim to contribute to the theoretical understanding of the prevention target to children exposed to the ACEs. This study offers insights for practitioners, policymakers, and educators, seeking to prevent EBPs in such at-risk preschool children.

## 2. Methods

### 2.1. Participants and Procedures

In September 2022, we randomly selected 11 kindergartens from both rural and urban areas in Wuhu City based on socioeconomic status and child density, recruiting newly enrolled 3-year-old children. Kindergarten principals and teachers were briefed about the study’s goals. We invited eligible children and their main caregivers to join the cohort study via the “WenJuanXing” platform. This study focused on non-disabled children to ensure more accurate results, excluding those with disabilities, as well as children whose parents had physical or mental health problems or communication difficulties. Parents were informed about the study’s purpose and procedures, as well as their right to withdraw at any time. After obtaining informed consent, they completed an online survey via the “WenJuanXing” platform, covering their children’s ACEs, PR, PMU, and their EBPs. In China, the academic year begins in September, and data for Time 1 (T1) were collected in the fall semester of the first academic year (September 2022). The second round and third round of data collection was conducted in September 2023 (T2) and September 2024 (T3). Initially, 839 parents provided data in September 2022, but 305 parent–child pairs were excluded during follow-up due to incomplete data or refusal to participate. The most common method of handling missing data, listwise deletion, is used when missing data meets the condition of being completely at random, resulting in unbiased estimates (Donner, 1982; Kang, 2013). Little’s MCAR tests confirmed the random pattern of missing data χ^2^ (67) = 63.15, *p* = 0.611. Ultimately, 534 parent–child dyads were included in the analysis. The current study received ethical approval from the affiliated university.

At baseline, the mean age of the children was 33.29 months. Of the children, 49.6% were boys (n = 265). Most of the parents obtained a vocational college degree or above. Most of the questionnaire responses were provided by the preschoolers’ mothers (n = 425, 79.6%), and others were reported by fathers (n = 109, 20.4%). The annual family income of the preschoolers varied from below CNY 50,000 to over CNY 300,000, with an average income between CNY 100,000 and CNY 150,000. Notably, 96.4% of families reported an income of at least CNY 50,000 per year, indicating that the economic status of our sample was higher than the average income level of poorer families in China. Descriptive statistics for all study variables can be found in Table 1.

### 2.2. Measures

ACEs: The Kaiser-CDC ACE Study questionnaire was applied to test children’s early adversities at age 3 (Felitti et al., 1998; Dube et al., 2002). The questionnaire was completed by the primary caregivers and was divided into three categories: household dysfunction (i.e., substance abuse, mental illness in household, parental separation or divorce), neglect (i.e., emotional neglect) and abuse (i.e., physical neglect). Each item was classified as 1 for “experienced” and 0 for “not experienced”. Zero to ten was the range of the cumulative ACEs score. The ACE questionnaire has been widely used to report early adversities by parents in Western countries and China (Zetino et al., 2020; Zhu et al., 2023). The ACE questionnaire had satisfactory internal consistency (Cronbach’s α = 0.73) in this current study.

Psychological resilience: Devereux Early Childhood Assessment for Preschoolers, Second Edition (DECA-P2), was used to examine preschoolers’ psychological resilience (LeBuffe & Naglieri, 2012). DECA-P2 has been widely used to test children’s psychological resilience worldwide (N. Yang et al., 2021; Carlson & Voris, 2018). Initiative (9 items, i.e., “keep trying when unsuccessful (show persistence).”), Self-Regulation (9 items, i.e., “handle frustration well.”), Attachment/Relationships (9 items, i.e., “seek help from children/adults when necessary.”), and Behavioral Concerns (11 items, i.e., “seem sad or unemotional at a happy occasion.”) are the four subscales that make up the DECA-P2 scale. Each item was scored on a 5-point Likert scale, with 0 representing never and 4 representing always. Psychological resilience was measured in this study using the attachment/relationships subscale, self-regulation subscale, and initiative subscale total score (Zhang et al., 2021). Raw scores were calculated into T-scores according to the manual. A higher total protective factor score indicated a higher level of resilience. Parent-reported DECA-P2 has shown good reliability and validity in Chinese children (Ding et al., 2023a). Cronbach’s α for the current study was 0.95.

Problematic media use: The problematic media use measure (PMUM), Chinese version, was employed to test children’s PMU (Domoff et al., 2019; Li et al., 2024). Tolerance and withdrawal (12 items; i.e., my child uses screen media for increasing amounts of time) and psychosocial problems (11 items; i.e., my child’s screen media use negatively affects his/her friendships) are the two subscales included in the PMUM. Primary caregivers rate their child’s behaviors during the previous month using 5-point Likert scale. The sum of the item scores and their division by 23 is used to produce the overall PMUM score. Higher PMU levels are indicated by higher scores. Cronbach’s α of the Chinese version of PMUM for our sample was 0.96.

Emotional and behavioral problems: Children’s behavioral and emotional problems were assessed using the Chinese version of the Strength and Difficulties Questionnaire (Goodman, 1997; Du et al., 2008). The children’s primary caregivers were asked to fill out the questionnaire, answering each question in light of their child’s behaviors during the preceding six months. Five scales make up the questionnaire: emotional problems (five items), hyperactivity (five items), peer problems (five items), conduct problems (five items), and prosocial behavior. A higher score on the prosocial behavior subscale exposes a child’s strengths, while the findings of the other four subscales are combined to produce a total score that represents difficulties. Each item was scored on a three-point scale ranging from 0 “not true” to 2 “certainly true,” for a total score of 0 to 50. In this study, we added the scores from the 4 subscales for difficulties to calculate the children’s total score for emotional and behavioral problems. A higher total score indicated a higher level of emotional and behavioral problems. The Cronbach’s α in our sample was 0.73.

Covariates: The child’s age (months), sex (0 = male, 1 = female), and family socioeconomic status (SES) were included as covariates based on prior research (Zhu et al., 2022) that indicated the significance of covariates when analyzing ACEs and children’s emotional and behavioral issues. We used the mother’s and father’s levels of education, as well as the family’s yearly income, to calculate the SES.

### 2.3. Data Analysis

First, descriptive analysis was used to present the demographic information of the participant. Pearson correlation analysis was performed to show the correlations between the main variables. Second, Macro Process 6 was used to test the two serial mediating effects of PR and PMU. ACEs were considered the predictor, PR and PMU were considered mediators, and children’s EBPs were regarded as the outcome. A total of 5000 bootstrap samples were used to estimate the 95% confidence intervals. A significant effect was indicated by a 95% confidence interval that did not include zero. The children’s age and sex and family’s SES were covariates in the data analysis. All statistical analyses were performed with SPSS 29.0.

## 3. Results

### 3.1. Descriptive Information

Table 1 presents the descriptive statistics for children’s age in months, sex, ACEs, PR, PMU, and EBPs. The means and standard deviations of the baseline year ages of the children were 33.29 years and 3.97 years, respectively. Of the children, 49.6% (n = 265) were boys, and 50.4% (n = 269) were girls. The average scores for ACEs (T1), PR (T1), PMU (T2), and EBPs (T3) were 0.40, 44.36, 1.62, and 8.20, respectively. Table 2 shows the bivariate correlations of the main variables.

### 3.2. The Serial Mediating Effects of Psychological Resilience and PMU

**Figure 1:**
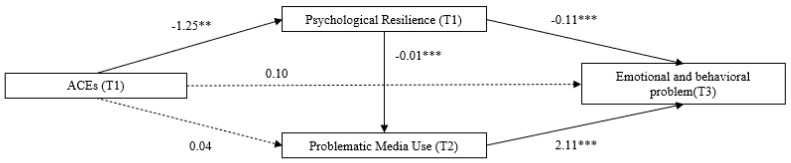
Serial mediating paths between adverse childhood experiences and emotional and behavioral problems via psychological resilience and problematic media use. ** *p* < 0.01, *** *p* < 0.001.

Table 3 and Figure 1 show the serial mediating roles of PR and PMU in the relationship between ACEs and EBPs in children. The results did not show significant associations between ACEs and children’s PMU and EBPs (b = 0.04, *p* > 0.05; b = 0.10, *p* > 0.05). In addition, Table 3 presents indirect paths. We found that ACEs were negatively related to PR at T1 (b = −1.25, *p* < 0.01), which in turn predicted children’s EBPs at T3 (b = −0.11, *p* < 0.001), which indicates ACEs can decrease the levels of PR, and PR can decrease the levels of EBPs. Furthermore, children’s PR at T1 negatively predicted PMU at T2 (b = −0.01, *p* < 0.001), which means PR may decrease the levels of PMU. We also found that PMU was significantly related to EBPs (b = 2.11, *p* < 0.001). Children with higher levels of PMU tend to have high levels of EBPs.

Table 4 shows the mediating effects. The mediating effect of PR was 0.132 (95% CI [0.051, 0.221]), indicating a significant indirect effect between ACEs and children’s EBPs. Children who experienced higher level of ACEs were more likely to have lower levels of PR, which in turn increases the risk of EBPs. The serial mediating effect via PR and PMU was 0.026 (95% CI [0.008, 0.054]), indicating a significant serial indirect effect of PR and PMU on the relationship between ACEs and children’s EBPs. ACEs may decrease the level of PR, which causes higher frequency of PMU and ultimately increases children’s EBPs.

## 4. Discussion

Despite warnings from researchers and pediatricians about the risks of ACEs in early childhood and evidence that ACEs in early childhood may be linked to various negative developmental effects (Jimenez et al., 2016; Bright & Thompson, 2018), few studies have investigated ACEs and their underlying mechanisms among Chinese preschoolers. This is the first longitudinal study to investigate PR and PMU as mediators in the relationship between ACEs and EBPs among Chinese preschoolers. In the proposed model, ACEs did not have a longitudinal direct effect on EBPs; however, two significant indirect pathways were identified. The results of this study may assist in the development of targeted interventions to prevent children’s early adversities.

Consistent with previous research (Wang et al., 2023), we did not find that ACEs directly predict children’s PMU or EBPs. According to Domoff et al. (2020), distal factors (such as ACEs), in themselves, do not convey an additive risk to children; rather, they indicate sensitivity or risk factors that, when combined with their effects on proximal factors, lead to the development of problematic media usage over time. Latent vulnerability theory (McCrory & Viding, 2015) suggests that the effects of ACEs may not appear immediately but rather emerge over time as stress sensitivity or emotional dysregulation systems develop. Moreover, physical aggression from parents is usually employed to enforce immediate compliance and may restrict children from media use (H. Yang et al., 2022). Çaylan et al. (2021) found that authoritarian parenting was not related to preschool children’s excessive screen time. In Chinese society, harsh parenting is often perceived not as abuse but as an expression of parental love, responsibility, and a method to guide children toward fulfilling their social, moral, and academic goals (J. Hou et al., 2011). Children may not always interpret such disciplinary actions as signs of parental rejection or excessive harshness and therefore may not develop EBPs as a result (Zhu et al., 2023). Similarly, Zhou et al. (2024) found no longitudinal effect of cumulative ACEs on children later internalizing or externalizing problems. They suggested that long-term mental health difficulties may arise from unresolved issues during earlier developmental stages, rather than being directly caused by early ACEs. Young children have high neuroplasticity, which means they are better able to heal and adapt to early adversity (Fox et al., 2010). Because of their developmental resilience, the consequences of ACEs may not emerge as stable PMU or EBPs in preschool children. With supportive surroundings (e.g., quality preschool), many children “bounce back,” which may reduce the long-term link between early ACEs and later maladaptive behaviors (Sciaraffa et al., 2018).

The results showed that PR mediates the relationship between ACEs and EBPs, which were similar with previous studies (Zhu et al., 2023). We found that ACEs in early childhood relate to the level of resilience decline, which causes an increase in behavioral problems. Resilience is a positive adaptation or the ability to cope with risk or adversity (Rutter, 2006). Children with high resilience are better able to deal with problems and unfavorable life situations (Gartland et al., 2019). Early brain development is especially vulnerable to toxic stress caused by ACEs; exposure to ACEs in the early years may damage the developmental architecture of the brain and influence children’s resilience development (Spenrath et al., 2011). According to ecosystem theory (Bronfenbrenner, 1979), a child requires at least one adult who genuinely cares for and supports them in order to develop resilience. It is difficult for children to become resilient if the prevalence of ACEs within the child’s home or ecological system is high (Sciaraffa et al., 2018). Moreover, children with lower levels of resilience have more mental health problems (Mesman et al., 2021). For example, children with higher PR tend to use reappraisal as an emotion management approach when they encounter emotional difficulties (J. Huang et al., 2023). Children with high resilience have positive cognition, and they tend to employ reappraisal to prevent negative emotion generation (Mak et al., 2011). Children with low levels of PR are more likely to show anxiety symptoms and aggressive behaviors (Holmes et al., 2015; Ding et al., 2023b).

Moreover, our findings also add to the new finding that PR and PMU serially mediate the relationship between ACEs and EBPs. Specifically, ACEs in early childhood could decrease children’s PR, which is associated with PMU and ultimately increases EBPs. Similar to previous research, children’s resilience was associated with PMU (Cao et al., 2022). According to IT-CPU theory (Domoff et al., 2020), early adversities and PR are distal and proximal factors that contribute to the development of PMU, and the increase in PMU later cause EBPs (Li et al., 2024). Adverse experiences in early childhood may affect children’s brain development and form insecurity attachment (Baer & Martinez, 2006), which may decrease the level of PR (Spenrath et al., 2011). Resilient children are better able to control their urges, are less likely to engage in risky actions, and exhibit fewer addictive behaviors (Hao et al., 2023). Children with high PR are less likely to develop undesirable situations (such as media addiction) because they can respond adaptively to difficult life events and participate in good behaviors and activities (X. L. Hou et al., 2017; Yam et al., 2024). Resilient children also can maintain positive bonds with their family (Herrman et al., 2011); however, children who experience psychological rejection, a lack of parental support, and indifference are more likely to spend more time on media (Xu et al., 2015). Previous studies have also suggested that children’s PMU was associated with EBPs. PMU in early childhood can predict children’s social–emotional development (Coyne et al., 2024; K. Choi & Hong, 2025). Hence, ACEs in early childhood can inhibit children’s PR and develop PMU, which in turn increases the possibility of EBPs.

## 5. Limitations and Future Research

The current study extends previous research by revealing an underlying mechanism of how ACEs in early childhood affect EBPs in children. A strength of this study is the use of a longitudinal mediation model to reveal whether PR and PMU indirectly affect ACEs and EBPs. Nevertheless, this study has some limitations. First, children’s ACEs were reported by their parents, and this study excluded children whose parents had mental health problems in order to ensure the accuracy of parent-reported answers to the questionnaire, which might have resulted in the exclusion of a core high-risk group for household dysfunction regarding ACEs, potentially underestimating the prevalence of ACEs and their true association with EBPs. Future research should use more precise methods (i.e., interviews) to record adverse experiences in early childhood. Second, previous studies have suggested that parenting styles could exacerbate children’s media dependency or emotional problems. We suggest that future research could add parenting styles (i.e., authoritarian parenting) to reveal environment–individual interaction mechanisms between ACEs, PR, PMU, and EBPs. Third, the sample was drawn from Wuhu city in China, which has a medium-sized national economic status. The results of the current study may also be affected by cultural factors (i.e., Confucian philosophies) and may not be generalizable to the entire population. We suggest that future research should focus on children’s early adversities from rural areas of China and other underrepresented populations. Finally, the data for the ACEs and PR were cross-sectional and may not have revealed the causal relationship between ACEs and PR. Future research should use a cross-lagged panel model or different timepoint measures to test the relationship between ACEs, PR, PMU, and EBPs.

## 6. Conclusions

This study expands our understanding of the longitudinal effect of early adversities on children’s EBPs and their underlying mechanisms. Our main finding suggests that ACEs in early childhood are significantly related to a lower level of PR, which in turn leads to PMU and ultimately increases risk of EBPs. To reduce children’s PMU and EBPs, our findings suggest that appropriate intervention programs should be provided to children who had exposure to ACEs, and trauma-informed educational programs should be incorporated to promote resilience during preschool age. Parents should utilize the “anti-addiction system for minors”, which was implemented by Chinese government in 2021, and adopt intensive media control strategies (e.g., parental monitoring devices) for children to attenuate the negative effect of PMU.

## Figures and Tables

**Table 1:** Descriptive statistics for all variables.

Variables	Category	*n* (%) or Mean ± SD
Child’s age at baseline year age (Month)		33.29 ± 3.97
Child’s sex	Male	265 (49.6)
	Female	269 (50.4)
Father’s education level	Primary school or below	1 (0.2)
	Middle school or below	39 (7.3)
	High school or vocational secondary school degree	66 (12.4)
	Vocational college degree	133 (24.9)
	Bachelor’s degree	234 (43.8)
	Master’s degree or above	61 (11.4)
Mother’s education level	Primary school or below	3 (0.6)
	Middle school or below	33 (6.2)
	High school or vocational secondary school degree	63 (11.8)
	Vocational college degree	150 (28.1)
	Bachelor’s degree	237 (44.4)
	Master’s degree or above	48 (9.0)
Annual family income	<50,000 RMB	19 (3.6)
	50,001–100,000 RMB	79 (14.8)
	100,001–150,000 RMB	137 (25.7)
	150,001–300,000 RMB	206 (38.6)
	>300,000 RMB	93 (17.4)
Adverse childhood experiences (T1)		0.40 ± 0.99
Psychological resilience (T1)		44.36 ± 10.00
Problematic media use (T2)		1.62 ± 0.60
Emotional and behavioral problems (T3)		8.20 ± 4.09

**Table 2:** Bivariate correlations among the main variables.

	1	2	3
1 Adverse childhood experiences (T1)	--		
2 Psychological resilience (T1)	−0.131 **	--	
3 Problematic media use (T2)	0.080	−0.183 **	--
4 Emotional and behavioral problems (T3)	0.085 *	−0.328 **	0.365 **

Note: * *p* < 0.05, ** *p* < 0.01.

**Table 3:** Mediating effects of psychological resilience and problematic media use on adverse childhood experiences and emotional and behavioral problems.

Predictors	Model 1 (Criterion PR)		Model 2 (Criterion PMU)		Model 3 (Criterion EBPs)	
	*b*	*T*	*b*	*t*	*b*	*t*
CO: Children’s age	0.07	0.57	−0.01	−0.24	−0.01	−0.10
CO: Children’s gender	1.41	1.66	−0.01	−0.26	−0.26	−0.81
CO: FEL	0.23	0.4	0.04	1.17	0.19	0.87
CO: MEL	0.95	1.58	−0.05	−1.40	−0.23	−1.01
CO: AFI	0.45	1.01	−0.02	−0.85	−0.20	−1.21
X: ACEs	−1.25	−2.88 **	0.04	1.38	0.1	0.67
ME: PR			−0.01	−3.83 ***	−0.11	−6.42 ***
ME: PMU					2.11	7.79 ***
*R* ^2^	0.04		0.04		0.21	
*F*	4.03 ***		3.58 **		17.41 ***	

Note: FEL = father’s education level; MEL = mother’s education level; AFI = annual family income; ACEs = adverse childhood experiences; PR = psychological resilience; PMU = problematic media use; EBPs = emotional and behavioral problems. ** *p* < 0.01, *** *p* < 0.001.

**Table 4:** Serial mediating paths between adverse childhood experiences and emotional and behavioral problems via psychological resilience and problematic media use.

	Effect	Boot SE	Boot LLCI	Boot ULCI
Direct effect				
ACEs → EBPs	0.109	0.163	−0.210	0.428
Indirect effects				
ACEs → PR → EBPs	0.132	0.044	0.051	0.221
ACEs → PMU → EBPs	0.076	0.054	−0.016	0.198
ACEs → PR → PMU → EBPs	0.026	0.012	0.008	0.054

Note: ACEs = adverse childhood experiences; PR = psychological resilience; PMU = problematic media use; EBPs = emotional and behavioral problems.

## Data Availability

The data are not publicly available due to privacy or ethical restrictions.
